# Team Emotional Intelligence in Working Contexts: Development and Validation of the Team-Trait Meta Mood Scale (T-TMMS)

**DOI:** 10.3389/fpsyg.2020.00893

**Published:** 2020-05-26

**Authors:** Aitor Aritzeta, Rosa Mindeguia, Goretti Soroa, Nekane Balluerka, Arantxa Gorostiaga, Unai Elorza, Jone Aliri

**Affiliations:** ^1^Department of Basic Psychological Processes and Development, University of the Basque Country UPV/EHU, San Sebastian, Spain; ^2^Department of Social Psychology and Behavioral Sciences Methods, University of the Basque Country UPV/EHU, San Sebastian, Spain; ^3^Department of Mechanic and Industrial Production, University of Mondragon, Mondragon, Spain

**Keywords:** emotional intelligence, teams, leadership, validity, reliability, scale

## Abstract

The collective construct of Team Emotional Intelligence (TEI) has been widely used and discussed. However, although several studies have examined the relationship between individual emotional intelligence and transformational leadership, few reports have explored the TEI of leadership teams. The aim of this study was to develop a scale to measure TEI, developing and validating the T-TMMS in a sample of 1,746 participants grouped into 152 leadership teams. The research design of the study was cross-sectional, and, in order to observe reliability as well as the construct, convergent, and predictive validity of the scale, we conducted an internal consistency analysis, confirmatory factor analysis, as well as a correlation and hierarchical linear regression analysis. The T-TMMS showed a three-factor structure (Attention, Clarity, and Repair), with adequate internal consistency, temporal stability, and convergent validity. We also examined the relationship between TEI and organizational performance. The limitations and implications of this new scale for organizational contexts are discussed.

## Introduction

The collective construct of Team Emotional Intelligence (TEI) has been widely used and discussed in the field of work and organizational psychology as an important predictor of a number of variables related to individual and group behavior. For example, it has been shown that teams with high TEI cooperate more, coordinate their work better, and communicate more effectively than do teams with low TEI (Lee and Wong, 2017). Studies have likewise found that teams composed of individuals with high EI showed lower levels of conflict, higher levels of cooperation, and better results in terms of team effectiveness and performance (Ghuman, 2016).

Despite these findings and the large number of studies regarding emotion in work contexts (e.g., Ashkanasy and Dorris, 2017), research on collective or group emotions in leadership teams is still scarce, and most studies of EI in leadership teams examine individual EI rather than the collective construct of TEI (Miao et al., 2016a, b). Likewise, although several studies have examined the relationship between individual EI and transformational leadership (López-Zafra et al., 2017), few reports have explored the TEI of leadership teams.

There are two approaches to examine TEI: a model based on individual variables – that is, data measured at an individual level are aggregated to a higher level (i.e., group or organization); and a model based on groups or teams; that is, variable measures that come from the groups or teams (i.e., group or team level information is examined). The individual-referent model proposes that TEI is an individual ability that members of a team can share, combine, and use when the team needs it, whereas the team-based model argues that TEI is a variable that is better measured examining team members’ perceptions about the team as a whole (Druskat et al., 2017).

These approaches have shown different associations with performance depending on the type of tasks examined. Team performance in low-interdependence tasks is related to each team member’s EI skills and to the possibility of summing them (Day et al., 2004), and individual-based EI models can probably better examine this type of individual competency (Rezvani et al., 2018). However, in high-interdependence tasks, as in those present in leadership teams, team performance depends on the whole team’s ability (Courtright et al., 2015). Top managerial teams, like leadership teams examined in this research, are teams centered on decision-making processes. These processes involve high emotionality and interdependence, and their performance might depend on the quality of the interactions between the teammates more than on their individual abilities. In this sense, Wei et al. (2016) reported that, in leadership teams, a team-referent measure of EI was the strongest predictor of emotion-related outcomes (i.e., conflict) in decision-making teams.

In the field of work and organizational psychology, only two measures of group EI have been proposed: the Workgroup Emotional Intelligence Profile (WEIP; Jordan et al., 2002) and the Group Emotional Intelligence (GEI) Survey (Druskat and Wolff, 2001). The WEIP is currently the only available tool for measuring group EI in Spanish-speaking work groups (López-Zafra et al., 2012). It evaluates the aggregated construct of the group EI using individual-referent model (instead of the collective construct), and it was adapted into Spanish using a sample of 332 employees, no leaders, belonging to 53 work groups.

Given the importance of group emotional phenomena for organizational contexts (Peñalver et al., 2017), the need to integrate variables across multiple levels of analysis so as to provide a more veridical account of leadership phenomena (Tse et al., 2018), and the scarcity of instruments for measuring group EI in Spanish-speaking leadership teams, the goal of this study was to develop a short and easy-to-administer questionnaire for measuring TEI in leadership teams using the team-referent model.

TEI can be considered an extension of a group’s ability to collaborate and work interdependently – in other words, its functional intelligence (Sternberg, 1984). Following Ashkanasy (2003), we regarded TEI as being more than the sum of individual emotional intelligence. If we accept that groups and teams may have and display emotions (van Kleef and Fischer, 2016), and also accept that leadership teams that work in a stable and constant over time manner can be understand as groups (Hawkins, 2017), then it is possible to expect different leadership teams to show different levels of TEI. Some processes, such as “emotional contagion” (Totterdell et al., 1998), “vicarious affect processes” (Fultz and Nielsen, 1993), and “interaction synchrony processes” (Siegman and Reynolds, 1983), support the idea that affective experiences located at the individual level may be aggregated to create a group-level affective construct.

Therefore, TEI can be considered as a construct located at the group level that is based on team members’ shared subjective emotional experiences. These shared subjective experiences contribute to the creation of a set of expected behaviors that influence an individual’s emotional experience (Druskat et al., 2017). The TEI examined here is a consequence of the nature of interaction occurring between leaders in the team. Such interaction generates a group construct (TEI) that is different from the individual EI traits that members of that team have. Thus, TEI can be considered a “collective construct” related to the team (Morgeson and Hofmann, 1999). More specifically, and consistent with the cognitive components of EI described by Salovey et al. (1995), the measure of TEI developed here examines the degree to which leaders in the same team consider that their team pays attention to and values the feelings of teammates, understands the emotions felt in the team, and uses positive thinking to repair negative moods in the team.

Other constructs regarding the collective emotional experiences as the group climate have also been shown to be clearly related to workers’ adaptive behavior. Encouraging group climates has been related to goal-oriented attitudes and behaviors. However, non-encouraging climates have been related to negative results, for example, avoidance conflict-managing behaviors or disruptive behaviors (Patrick et al., 2003). In this same line, it has been observed that workers belonging to teams with high involvement, that is, teams that show motivation to learn and high levels of member’s identification, also show higher experiences of flow (Csikszentmihalyi and Csikszentmihalyi, 1988; Chang et al., 2018) and a greater ability to examine reality from the perspective of others (Flinchbaugh et al., 2016) compared to workers belonging to teams with low levels of involvement (Pekrun et al., 2007).

Taken together, these findings suggest that team members in work contexts, such as leadership teams, may pay special attention to the feelings and emotions they perceive while interacting with others in the team. Furthermore, these interactions may have an important influence on workers’ behaviors toward both the organization and each other (Li et al., 2017). In the Spanish-speaking context, however, there is currently no short, easy-to-administer, reliable, and valid instrument for examining these kinds of emotional interactions from the perspective of Mayer and Salovey’s (1997) model of EI.

Taking into account the aforementioned, the aim of this study was to develop and validate a short and easy-to-administer scale called T-TMMS to measure TEI in leadership teams.

## Materials and Methods

### Development of the T-TMMS

Many investigations into the realm of EI have been interested in the elaboration of scales for measuring individual EI (Extremera et al., 2009). In this context, one of the most widespread theoretical models is that proposed by Mayer and Salovey (1997), and one of the most widely used instruments for measuring perceived EI is the Trait Meta-Mood Scale (TMMS; Salovey et al., 1995). The TMMS measures “individual beliefs about the importance of paying attention to one’s own emotions and feelings” (Attention), “about the capacity for understanding one’s own emotions” (Clarity), and “about the ability to repair negative emotional states and maintain positive ones” (Repair). Fernández-Berrocal et al. (2004) adapted the original TMMS into Spanish, producing an abridged version comprising 24 items. Based on this model, a collective measure known as the G-TMMS was recently developed to examine group EI in school classrooms (Aritzeta et al., 2016). In the present study, the items for assessing team EI were derived from those featured in the G-TMMS, and hence we called the new measure the T-TMMS.

As in the development of the G-TMMS the process of creating the T-TMMS used the “consensus-based change-of-reference” strategy, following Chan’s (1998) theory of group-level composition models. This strategy supports the idea that a group-level characteristic can be examined by changing the reference from the individual to the group level; that is to say, changing the framework of the tapped characteristic from the individual to the group level. Additionally, the within-group agreement should be ensured by means of the James inter-coder reliability index (James et al., 1993). In the G-TMMS and following the aforementioned “consensus-based change-of-reference” process, the reference framework for responding to items was changed from the individual self-evaluation (e.g., “I pay a great deal of attention to my feelings”) to the perception of classroom experience (e.g., “In this classroom we pay a great deal of attention to our feelings”). In developing the T-TMMS, the classroom framework was changed to the leadership team framework (e.g., “In this team we are able to describe our feelings”). Taking into account that a short, 12-item version of the TMMS has previously been validated (Salguero et al., 2009), the initial version of the T-TMMS included 12 items, each with a 6-point Likert-type response format anchored by “Strongly disagree” and “Strongly agree.” These items measure the degree to which, on average, leaders or workers belonging to a stable team perceive that their team attends to feelings and values them, is clear rather than confused about feelings, and adopts positive thinking to repair negative group moods.

Two psychologists specialized in teamwork and emotions independently modified the reference framework from the classroom to the team level. Both psychologists were familiar with the fundamental psychometric criteria of item construction. Before reaching an agreement on all items, each item of each version was examined, paying special attention to “team” as the key reference framework.

### Participants

The study sample comprised 1,764 leaders grouped into 152 business teams: 40.3% from the food distribution sector (stores analyzed in the present research fell under the Small and Medium-sized Enterprises (SME) category with an average headcount of 100 people per store), 14.2% from the education sector (schools analyzed here also are small with less than 100 workers), 27.4% from the industry sector (72% of them were SME and 28% were large enterprises), and 18% from the service sector (all of them were small firms). A total of 38% of the total sample was female, and the average age was 42 (*SD* = 8.68). All the firms are located in the Basque Country (northern Spain) and are part of the Mondragon Cooperative Corporation, sharing four corporate values: cooperation, participation, social responsibility, and innovation. All organizations were private and cooperatives being with small to average sizes.

### Data Collection Procedure

Data were collected between 2013 and 2017, after having agreed the conditions with the directors of each participating firm. Data was obtained through survey questionnaires that were completed voluntarily by managers. Data was gathered through surveys completed both on paper and electronically (i.e., managers received the survey through the email). In both formats (i.e., paper or email) subjects were briefly informed that the study pertained to how they felt about their job environment, their workmates, and the company they worked for. They were asked to answer with sincerity, and absolute confidentiality was guaranteed. On the occasions where managers completed the survey on paper, specific dates and schedules were agreed with the companies, and suitable rooms were made available. Informed consent was requested from each participating group. The response rate was 93%.

The study fulfiled the standards of the Ethics Committee for Research Involving Humans of the University of Mondragón (ID: Bateratzen-Partaidetza-IGB-39).

### Other Instruments and Measures Used to Validate the T-TMMS

#### Workgroup Emotional Intelligence Profile (WEIP-3)

Evidence of convergent validity for the T-TMMS was sought by applying the WEIP-3 (Jordan et al., 2002) in its Spanish version (López-Zafra et al., 2012). The WEIP-3 was chosen as it is based on Salovey and Mayer’s (1990) original construct of EI, the same theoretical model from which the T-TMMS is derived.

The WEIP-3 analyses perceived EI in workgroups. Its 27 items, each rated on a 7-point Likert scale, measure seven aspects organized into two broad dimensions: (a) the “ability to deal with one’s own emotions;” and (b) the “ability to deal with the emotions of others.”

Three of the seven aspects were considered for the present study: (a) *Awareness*: awareness of emotions (e.g., “I am aware of my feelings when working with my teammates”); (b) *Expression*: ability to discuss/articulate emotions (e.g., “I can explain the emotions I feel to my team”); and (c) *Management*: ability to use one’s own emotions to facilitate thinking (e.g., “When I am angry with a member of my team I can overcome that emotion quickly”). The scale has shown good reliability and validity (Jordan et al., 2002), and, in its Spanish version, values were 0.92 (Awareness), 0.83 (Expression), and 0.89 (Management).

#### Group Emotional Intelligence (GEI) Survey

The Group Emotional Intelligence (GEI) Survey (Wolff, 2006) is based on Goleman’s (1995) framework of awareness and regulation of emotion at multiple levels. The GEI Survey examines six dimensions (Group awareness of members, Group management of members, Group self-awareness, Group self-management, Group social awareness, and Group management of external relationships) and nine norms associated with these dimensions. For this study we used two norms associated with the dimension of group self-management: *creating resources for working with emotions*, which implies accepting emotions as part of a group and encouraging the expression and examination of feelings (e.g., “When there is tension in our group we acknowledge or discuss it”), and *creating an affirmative environment*, which is associated with creating a positive group affect and an optimistic outlook (e.g., “In our group, we are optimistic about our ability to deal with challenges”). The scale has shown good validity, as the reliabilities of all norms ranged from 0.88 to 0.74 (Druskat and Wolff, 2001).

### Data Analysis

In order to examine the dimensionality of the T-TMMS we conducted several Confirmatory Factor Analyses (CFAs). The estimation method used was Maximum Likelihood (ML), which is the most widely used estimator in applied CFA. This estimator has been recommended for data under the assumption that (1) there is a large sample size, (2) the indicators of the factors have been measured on continuous scales, and (3) the distribution of the indicators is normal (see Table 1 for data distribution results) (Brown, 2015). The goodness-of-fit indices were the Comparative Fit Index (CFI), the Tucker-Lewis Index (TLI), and the Root Mean Square Error of Approximation (RMSEA). In the case of the CFI and TLI, values above 0.90 and 0.95 indicated an acceptable and excellent fit, respectively. For the RMSEA, values below 0.08 indicated an acceptable fit, and values less than 0.06 indicated a good fit (Hu and Bentler, 1999; Brown, 2015).

**TABLE 1:** Initial items included in the T-TMMS, their descriptive statistics, and factor loadings.

Items	*M*	*SD*	*s*	*k*	Attention	Clarity	Repair
1. In my team we usually care about what our workmates are feeling	4.81	0.74	–1.06	1.84	0.658		
2. In this team we are able to describe our feelings	4.38	1.07	–0.74	0.34		0.782	
3. Although we may feel sad at times, we have a positive outlook as a team	4.69	0.91	–0.55	0.10			0.811
*4. In this team we believe that it is worth paying attention to work-mates’ feelings (Eliminated)*	4.77	0.89	–1.04	1.43	*0.282*		
5. We usually know how our teammates feel	4.27	0.84	–0.61	0.25		0.739	
6. Although we might feel bad, all team members try to have a positive outlook	4.60	0.93	–0.58	0.29			0.855
7. In this team most of us know what our mood is at any given moment	4.32	0.76	–0.30	0.17	0.714		
*8. In this team we normally know what we feel about our teammates (Eliminated)*	4.33	0.89	–0.57	0.85		*0.154*	
9. In this team we try to have a positive mood	4.86	0.75	–0.84	1.37			0.857
10. We often think about what feelings our teammates might have	4.50	1.02	–0.67	0.29	0.823		
11. We usually know what we feel in different situations	4.41	0.63	–0.75	0.75		0.714	
*12. When we are angry we try to change our mood (Eliminated)*	4.49	0.80	–0.48	0.66			*0.466*

**M, mean; SD, Standard deviation; s, skewness; k, kurtosis. The items shown here are English translations of the Spanish version administered in this study. The original Spanish items are available from the authors*.*

Internal consistency of the T-TMMS was estimated by means of Cronbach’s alpha, omega and hierarchical omega coefficients and Average Variance Extracted (AVE) and composite reliability for each subscale was calculated for reliability. To ensure that mean scores adequately represented emotional intelligence at the team level (i.e., TEI), the James indices of inter-coder reliability (James et al., 1993) were used. Besides, temporal stability was assessed using the test-retest procedure, with the instrument being re-administered to a smaller sample. We used 32 teams (21% of the total sample) comprising a total of 241 leaders. This sample size was established using power analysis to establish the minimum sample needed for a power of 0.95 and 0.05 alpha value. Following the time-lap criteria of previous investigations, the instrument was re-administered 8 weeks after the initial data collection. In order to obtain evidence of the instrument’s convergent and discriminant validity, we calculated Pearson correlation coefficients between mean team scores on the T-TMMS subscales and scores on the WEIP and GEI subscales. Finally, hierarchical regression analysis was conducted to evidence the predictive validity of the new scale.

## Results

### Descriptive Statistics

The descriptive statistics of all items of the T-TMMS scales are shown in Table 1. Afterward, Kim’s (2013) normality was assessed based on the skewness and kurtosis values (Kim, 2013). The recommended cut-off values of 2.0 for skewness and 7.0 for kurtosis (Curran et al., 1996) were applied. All items fell within the cutoff values, meaning that our data were normally distributed.

### Confirmatory Factor Analysis

We began by testing a three-factor solution (Attention, Clarity, and Repair) with four items corresponding to each factor. As this initial model did not yield an adequate fit (χ^2^_51_ = 1371.003, *p* = 0.0001, CFI = 0.90, TLI = 0.87, RMSEA = 0.12, 90% CI = 0.11–0.13), we analyzed the factor loadings (see Table 1) and eliminated those items with loadings below 0.50 (items 4, 8, and 12). In addition, the three-factor solution was compared with the unifactorial solution of the scale (χ^2^_27_ = 1193.76, *p* = 0.0001, CFI = 0.87, TLI = 0.83, RMSEA = 0.16, 90% CI = 0.15–0.16). The final model showed better fit than the unifactorial model (χ^2^_23_ = 234.015, *p* = 0.0001, CFI = 0.98, TLI = 0.96, RMSEA = 0.07, 90% CI = 0.06–0.08) with adequate factor loadings (see Table 2 for the final model).

**TABLE 2:** Final 9-item version of the T-TMMS after eliminating the problematic ones.

Items	Attention	Clarity	Repair	Total
1. In my team we usually care about what our workmates are feeling	0.63			
2. In this team we are able to describe our feelings		0.77		
3. Although we may feel sad at times, we have a positive outlook as a team			0.83	
5. We usually know how our teammates feel		0.76		
6. Although we might feel bad, all team members try to have a positive outlook			0.87	
7. In this team most of us know what our mood is at any given moment	0.69			
9. In this team we try to have a positive mood			0.85	
10. We often think about what feelings our teammates might have	0.85			
11. We usually know what we feel in different situations		0.75		
α [CI]	0.77 [0.76, 0.79]	0.81 [0.80, 0.82]	0.88 [0.87, 0.89]	0.92 [0.91, 0.92]
ω [CI]	0.76 [0.77, 0.80]	0.82 [0.81, 0.83]	0.89 [0.88, 0.89]	0.92 [0.91, 0.92]
ωh	0.76	0.81	0.88	0.82
AVE	0.53	0.58	0.72	−
CR	0.76	0.81	0.88	−

**The items shown here are English translations of the Spanish version administered in this study. The original Spanish items are available from the authors. α, alpha; ω, omega; ω; h, hierarchical omega; CI, confident intervals 95%; AVE, Average Variance Extracted; CR, Composite Reliability.**

### Reliability

The T-TMMS showed adequate internal consistency, exceeding the cut-off value of 0.75 that is generally accepted for instruments in the area of health sciences for Cronbach’s alpha and omega (Viladrich et al., 2017). All the values were also above the desired cut-off value of 0.5 in the case of AVE and 0.7 (Henseler et al., 2015) in the case of composite reliability. Please, see Table 2 to observe internal consistency and reliability values.

In order to examine the temporal stability of the T-TMMS, we calculated the correlation index between mean team scores at the two assessment points, obtaining a value of 0.86. James indices of inter-coder reliability (James et al., 1993) had previously been calculated to ensure that these mean scores adequately represented emotional intelligence at the team level (i.e., TEI). The indices ranged between 0.80 and 0.98, suggesting that the leaders belonging to each team had similar perceptions about the construct that the instrument sought to measure.

### Convergent and Discriminant Validity

As in the case of reliability, James indices of inter-coder reliability were calculated for scores on the WEIP and GEI subscales prior to estimating correlation coefficients. Values ranged from 0.79 to 0.85 for the WEIP and between 0.83 and 0.93 for the GEI subscales. Pearson correlation coefficients between T-TMMS scores and scores on the WEIP and GEI subscales are shown in Table 3. Even the correlation between Attention and Clarity dimensions of the T-TMMS scale (*r* = 0.80; *p* < 0.01.) were slightly high following the Brown (2015) criterion, and it can be considered that the three dimensions showed an adequate discriminant validity and are not overlapping.

**TABLE 3:** Pearson correlation coefficients between T-TMMS scores and scores on the WEIP and GEI subscales.

	1	2	3	4	5	6	7	8
1. T-TMMS attention	**−**	0.801**	0.679**	0.601**	0.494**	0.721**	0.697**	0.582**
2. T-TMMS clarity		**−**	0.654**	0.651**	0.551**	0.735**	0.689**	0.555**
3. T-TMMS repair			**−**	0.662**	0.482**	0.651**	0.558**	0.725**
4. WEIP management				**−**	0.591**	0.730**	0.606**	0.574**
5. WEIP expression					**−**	0.627**	0.620**	0.572**
6. WEIP awareness						**−**	0.699**	0.678**
7. GEI-WE							**−**	0.575**
8. GEI-CA								**−**

**p < 0.01. GEI-WE, Group Emotional Intelligence Survey, “Working with emotions” norm; GEI-CA, Group Emotional Intelligence Survey, “Creating an affirmative environment” norm.

### Predictive Validity

In order to examine the predictive validity of the new scale, we analyzed the ability of the TEI to predict group cohesion using linear regression analysis. In addition, we compared the predictive power of T-TMMS and WEIP when predicting group cohesion. Considering the type of teams participating in this study and the aggregating model behind each scale, we expected T-TMMS would explain strongly group cohesion than WEIP-3. The results indicated that the percentage of the variance explained by T-TMMS (*R*^2^ = 0.21) was bigger than the explained by WEIP (*R*^2^ = 0.15).

To better understand the differences among team EI measures, we conducted a series of hierarchical regression analyses. The focal (the T-TMMS) and the alternative (WEIP) variables were entered in to the first and the second steps of the regression model. The order of entry was subsequently reversed.

In their associations with team cohesion, as illustrated in Table 4, the T-TMMS explained additional variance in team cohesion beyond the explaining capacity of the WEIP-3. When the order was reversed, however, the WEIP-3 did not explain additional variance.

**TABLE 4:** Regression results for testing the incremental validity of T-TMMS and WEIP-3 measures.

Normal order				Reversed order			
Variable added	B	*R*^2^	Δ*R*^2^	Variable added	β	*R*^2^	Δ*R*^2^
Criterion: team cohesion							
T-TMMS TEI	0.441**	0.192		WEIP_3 TEI	0.381**	0.142	
WEIP-3 EI	0.120	0.195	0.003	T-TMMS TEI	0.352**	0.195	0.05

**p < 0.01.

## Discussion

The goal of this study was to develop a valid and reliable questionnaire for measuring perceived TEI in leadership teams. The T-TMMS was shown to have adequate psychometric properties and replicated the three-factor structure (i.e., Attention, Clarity, and Repair) of both the original TMMS (Salovey et al., 1995) and its Spanish adaptation (Fernández-Berrocal et al., 2004).

The reliability of the T-TMMS was supported by the indices obtained for internal consistency and temporal stability. Evidence of convergent validity was provided by correlations between T-TMMS scores and scores on the WEIP and GEI subscales with only one exception – the moderate correlation between the T-TMMS and the Expression subscale of the WEIP. Whereas the other WEIP dimensions examine individual emotional behavior when interacting with groups, the Expression dimension focuses on emotional communication during this interaction (in this case, with the team), which is more difficult to assess than is behavior. This is one possible reason to explain the observed moderate correlation. As expected, the TEI construct was positively correlated with two of the WEIP subscales and with the GEI subscales. Given the results for convergent validity, the T-TMMS can be considered a useful instrument for the assessment of group emotional processes.

Regarding the predictive validity of T-TMMS, the scale was shown to be a stronger predictor of team cohesion than the individual referent measure used and demonstrated incremental validity over the WEIP. This result is consistent with the research of Wei et al. (2016) who reported that team-referent measure of EI was the strongest predictor of emotion-related outcomes (i.e., conflict) in teams with high interdependence tasks.

If we wish to explain and predict how work teams function, it is important to consider in a holistic way how workers’ emotions, cognitions, and motivations may result from their interactions within a team. Indeed, being part of a work team implies a complex combination of information processing and emotional responding that could influence team members’ responses, as the same worker may experience different emotional responses to a dramatic event in two different teams, which, in turn, might influence a worker’s perceptions of TEI (Ghuman, 2016). In this context, the T-TMMS can be regarded as a useful instrument for assessing group emotional processes, and its application could help to highlight the importance of leader relationships for the development of emotional wellbeing in teams. As research has shown, organizations characterized by better relationships between workers and that are able to improve team performances and reduce conflict are those that implement social and emotional learning programs (Menges and Bruch, 2009).

Some limitations should also be mentioned. The results should be generalized with caution, as the study sample was drawn exclusively from leaders of the Mondragon Cooperative Corporation located in Basque Country (Spain). Because of the specific characteristics of these kinds of corporations, future research should seek to replicate the findings obtained here in other organizational contexts and also in other countries. The relationships we observed also need to be examined longitudinally. A further limitation is that the results are based on self-report data, which may produce some bias and that the design of the study is cross-sectional. Future studies should therefore employ more objective measures and longitudinal research designs to verify the impact of TEI in organizations.

Despite these limitations, the T-TMMS can contribute to a better understanding of differences between leadership teams. Not only does it constitute an important addition to ability measures of EI, it should also help researchers and practitioners to assess whether the emotional context is likely to promote or impede an individual’s awareness of his or her emotional abilities and behavior. Furthermore, the T-TMMS could be used to explore inter-group differences in EI, providing information that would be useful for designing programs to increase group emotional intelligence.

## Data Availability Statement

The datasets generated for this study are available on request to the corresponding author.

## Ethics Statement

The studies involving human participants were reviewed and approved by Ethics Committee of the University of Mondragon. The patients/participants provided their written informed consent to participate in this study.

## Author Contributions

AA together with GS and RM were responsible of developing the theoretical foundations of the manuscript (Introduction, Discussion, and Conclusion). NB together with AG and JA were responsible of the methodological part of the manuscript, especially of the statistical analysis. Finally, UE was responsible of the process for gathering data and reviewing the manuscript.

## Conflict of Interest

The authors declare that the research was conducted in the absence of any commercial or financial relationships that could be construed as a potential conflict of interest.
